# Relationship between mental health and substance abuse on COVID-19 vaccine hesitancy in youth: A mixed methods longitudinal cohort study

**DOI:** 10.1371/journal.pone.0313157

**Published:** 2025-01-08

**Authors:** Louis Everest, Joanna Henderson, Clement Ma, Matthew Prebeg, Jacqueline Relihan, Lisa D. Hawke

**Affiliations:** 1 Centre for Addiction and Mental Health, Toronto, Ontario, Canada; 2 University of Toronto, Toronto, Ontario, Canada; Xiamen University - Malaysia Campus: Xiamen University - Malaysia, MALAYSIA

## Abstract

**Background:**

Mental health and substance use challenges are highly correlated in youth and have been speculated to be associated with COVID-19 vaccine hesitancy. Literature has also suggested that mental health challenges in youth have increased during the COVID-19 pandemic. However, the longitudinal relationship between mental health challenges in youth and COVID-19 vaccine hesitancy is not well established.

**Objective:**

We examined the relationship between mental health, substance use and COVID-19 vaccine hesitancy in youth during the COVID-19 pandemic.

**Methods:**

Youth ages 14 to 29-years participated in a longitudinal survey study. Participants provided sociodemographic, mental health, and substance use data, as well as qualitative and quantitative information on their vaccine perspectives every two months between February 2021 to August 2021, and on February 2022. Generalized estimating equation logistic regression models were used to analyze the effect of mental health and substance use on vaccine hesitancy over time. Qualitative content area analyses were used to identify trends in vaccine attitudes.

**Results:**

Mental health challenges and substance use frequency were associated with vaccine hesitancy, and significantly increased the odds of vaccine hesitancy over time. Additionally, mental health challenges were associated with decreases in vaccine hesitancy (OR: 0.80 (95% CI 0.66, 0.97)) when vaccines first began to emerge, but increases in vaccine hesitancy (OR: 1.72 (95% CI 1.32, 2.26)) one year later. Participants reported perceptions regarding vaccine safety and efficacy were the primary determinants influencing hesitant, uncertain, and acceptant vaccine attitudes. Additionally, changes in vaccine attitudes over time for some participants, were associated with changes in mental health.

**Conclusions:**

Increases in mental health challenges and substance use were associated with increases in COVID-19 vaccine hesitancy in youth over the COVID-19 pandemic. Health policy agencies should be aware of the potential impact of mental health challenges and substance use in youth, when developing vaccine policy and programs.

## Introduction

Recently, concerns with respect to COVID-19 vaccine hesitancy have become a topical discussion in literature [1–12]. ‘Vaccine hesitancy’ may be generally defined as the ‘delay in acceptance of the vaccine despite the availability of vaccination services’ [13]. Additionally, vaccine hesitancy has been acknowledged as representing a spectrum of beliefs, and may be informed based on a complicated interactions of socio-cultural, political, systemic, and historical determinants [2, 10, 11, 13]. Importantly, emerging research has suggested that racial, economic, and age-based disparities may exist regarding COVID-19 vaccine hesitancy [3, 14, 15]. However, there is currently a paucity of research examining long-term vaccine behaviours and perspectives in youth populations, despite their observed higher propensity for vaccine hesitancy [9, 16].

The negative impact of the COVID-19 pandemic with respect to the mental health of youth is relatively well established in literature [7, 16–18]. Specifically, studies have suggested that youth aged 14 to 28 with and without preexisting mental health challenges experienced increases in mental health concerns during the pandemic, and that such negative mental health impacts may be more severe in youth populations, compared to adult populations [16, 19]. Mental health concerns identified in youth include increased prevalence of anxiety and depression during the pandemic, among other conditions [20, 21]. Additionally, severe mental illness has been associated with an increased propensity of COVID-19-related morbidity and mortality [6]. Further, mental health and substance use challenges have been observed in prior literature to be highly correlated and interconnected [22]. These disparities in youth populations have been speculated to potentially be based on mental health-related developmental milestones that may typically first emerge during late adolescence and early adulthood, such as schooling, workforce engagement, early career development, relationship development, and autonomous living and decision-making, have been disrupted or altered by public health guidelines [16]. Additionally, prior literature has suggested COVID-19 has a negative impact on the overall health-related quality of life of adolescent and youth populations [23] (with the exception of sleep patterns in adolescent populations) [24]. This general trend of reduction in quality of life during the COVID-19 pandemic is consistent with those observed in adult populations [25].

Novel research has suggested that vaccine hesitancy may be potentially associated with mental health challenges during the COVID-19 pandemic [8, 17, 20, 21, 26]. Additionally, this relationship has been speculated to potentially impact youth populations more acutely compared to older populations, based on the higher observed propensity of vaccine hesitancy and mental health challenges during the pandemic in youth [7, 27]. However, this association is not well established in youth, and prior studies in general populations have suggested heterogeneous results [8, 17, 28]. Specifically, Moscardino et al. (2022) identified no differences in vaccine hesitancy between young adults with and without depressive symptoms, while Palgi et al. (2021) reported vaccine hesitancy was correlated with mental health morbidity in adults, and Murphy et al. (2021) reported that history of mental illness was protective for vaccine hesitancy in adults. Further, Farcas et al. (2022) identified 15 articles in a scoping review examining COVID-19 vaccine hesitancy in the psychiatric population. Specifically, Farcas et al. reported common risk factors for COVID-19 vaccine hesitancy included diagnosis of severe mental illness, substance use disorders, and younger age, among others [29].

Importantly, because prior studies examining vaccine hesitancy and mental health and substance use challenges used different objectives, different search strategies, and methodologies, it is difficult to draw general conclusions from their findings [12]. Additionally, as present literature has examined this association based on a case-control methodology, we cannot make inferences based on the temporality of the mental health and vaccine hesitancy relationship [12]. However, concerns in literature have been presented suggesting that the observed increases in mental health challenges in youth over the COVID-19 pandemic may potentially be associated with increases in propensity of vaccine hesitancy in youth [6]. Further, substance use disorders are well established to be highly comorbid with mental health challenges, and commonly emerge during adolescence and young adulthood [16]. However, the impact of substance use disorders with respect to COVID-19 vaccine hesitancy in youth is also not well established in literature [16]. Therefore, updated longitudinal studies examining the putative relationship between mental health and COVID-19 vaccine attitudes in youth populations, may represent a gap in the current literature [6].

Importantly, qualitative literature has also identified adolescent mental well-being as a potential priority topic area for future research examining vaccine hesitancy [30]. This putative relationship is based on the observation that symptoms associated with mental health challenges in youth populations reported during the COVID-19 pandemic may interact with behaviours associated with vaccine hesitancy [9, 30, 31]. For example, people living with mental health disorders such as obsessive-compulsive disorder may be hesitant to receive vaccines based on a fear of getting infected/injected with germs [9]. Additionally, people experiencing paranoid delusions may not trust the safety and efficacy of the vaccine or may endorse conspiracy theories [9]. Therefore, the putative relationship between mental health and vaccine hesitancy may be potentially understood as including multiple discrete pathways, that may represent heterogeneous magnitudes of effect [9, 18]. However, these speculated pathways are based on qualitative research in adult populations, which may potentially not represent the lived experience of youth with mental health challenges. Importantly, additional research is required to examine mental health and vaccine hesitancy both quantitatively and qualitatively in youth populations. Currently, discussion exists regarding if people with mental health challenges may be considered a priority vaccination group, in order allow this population earlier access to booster vaccines [27, 32]. However, the present study identified that although participants cited concerns regarding vaccine availability when vaccine first became available, increases in vaccine hesitant attitudes was the main determinant for participants not wanting to receive booster vaccines. Therefore, the rationale of the present study was to address this gap in literature and provide data for the consideration of youth with mental health and substance use challenges as a potential priority and targeted COVID-19 vaccination group.

The present study, conducted in Canada, reports results from a longitudinal cohort study, in which we examined changes in vaccine attitudes with respect to mental health and substance use challenges over the COVID-19 pandemic. Additionally, the present study is a youth-engaged project and was designed based on the Strategy for Patient-Oriented Research. Therefore, the present study utilized the guiding paradigm of pragmatism in a constructivist epistemology [33]. Specifically, this paradigm examined lived experience as paramount, and valued equally to research experience. Additionally, this paradigm was applied conjointly with the Patient Engagement in Research conceptual framework [34], to facilitate and guide engagement activities throughout the present study. The major constructs examined in the present study included vaccine hesitancy, mental health, or substance use.

The potential association between mental health challenges and vaccine hesitancy in youth has generated concerns in literature because this relationship may suggest that a population that appears to have a heightened risk of acquiring a COVID-19 infection, also potentially appears to have a heightened risk of hesitancy towards the COVID-19 vaccine [30, 32]. Additionally, based on the observed increases in mental health challenges in youth over the COVID-19 pandemic, this putative relationship may also suggest that vaccine hesitancy in youth with mental health challenges may have increased over the pandemic [30, 32]. However, a plurality recent studies have noted the absence of longitudinal studies and called for additional research examining this potential relationship [6, 9, 28]. Therefore, the objective of the present study is to examine the relationship between mental health, substance use and COVID-19 vaccine hesitancy in youth during the COVID-19 pandemic. Importantly, the aim of the present study was hypothesis generating, and identify topics and trends for future research.

## Methods

The present study implements a longitudinal cohort design. The present study examines self-reported mental health, substance use, and COVID-19 vaccine attitudes among youth.

**Fig 1:**
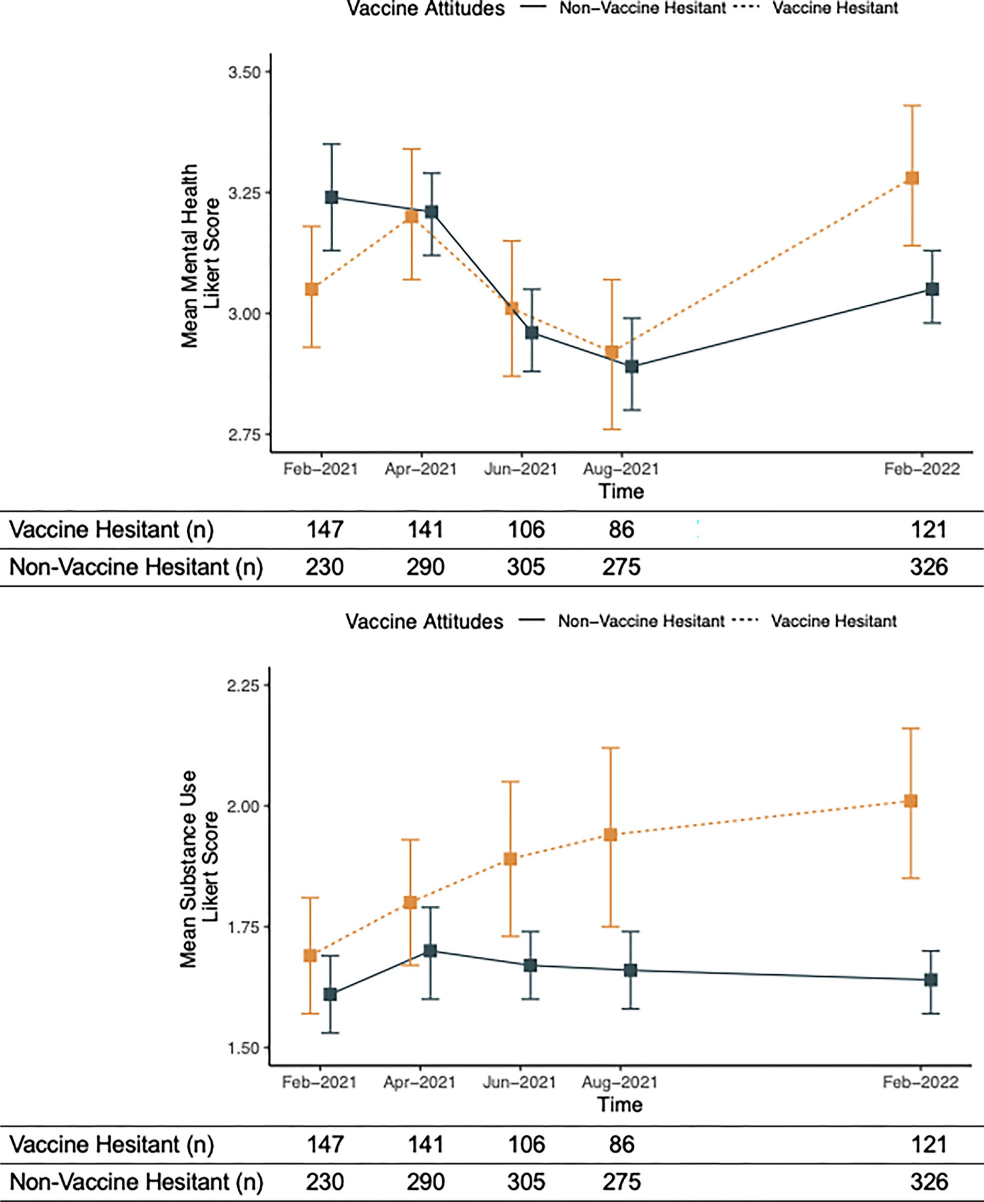
a: Descriptive means of mental health scores in vaccine hesitant and non-vaccine hesitant over the time. Solid grey bars: non-vaccine hesitant. Dashed orange bars: vaccine hesitant. Error bars represent 95% confidence intervals. Higher mean domain Likert scores represent increased mental health challenges. b: Descriptive means of substance use scores in vaccine hesitant and non-vaccine hesitant over the time. Solid grey bars: non-vaccine hesitant. Dashed orange bars: vaccine hesitant. Error bars represent 95% confidence intervals. Higher mean domain Likert scores represent increased substance use frequency.

The study participant sample and procedures have been previously reported in other related manuscripts [16, 35]. A consort diagram of included participants is presented in Fig 1 of the S1 Appendix. Briefly, youth ages 14 to 29-years were eligible for inclusion in the present study. Previously, this cohort was identified not to be representative of the Canadian population, and some diverse youth may not have been reached, for example, those without internet access [16]. Therefore, potential selection bias may have occurred in the present cohort. Participants were recruited between 8 and 29 April 2020, and electronic questionnaires were sent to participants every 2-months, from April 2020 to August 2021, and in February 2022, using email linking to the surveys on REDCap software [36]. The present analysis examined the period of February 2021 to February 2022 inclusive. In the present study, the same cohort of participants were followed longitudinally and were asked to report vaccine hesitancy information at least once during the examined study period in order to be eligible for inclusion.

The study population was recruited from four prior studies conducted out of the Centre for Addiction and Mental Health (CAMH) in Toronto, Ontario, Canada.

The NIMH-developed CRISIS tool Youth Self-Report Baseline version 0.3 was used. This tool has been externally validated and found to identify reproducible pandemic-related mood states [37]. Scales retained for the current study included perceived mental health and substance use during the past 2-weeks. Perceived mental health was defined based on the ten mental health items, including questions regarding mood and anxiety, sadness, enjoyment, irritability, and concentration issues, among others. Additionally, perceived substance use was defined based on frequency of use of substances including alcohol, cannabis, and opiates among others. Further, demographic information captured included ethnic origin/background, gender, age, highest level of education, and employment status at each time point examined.

**Fig 2:**
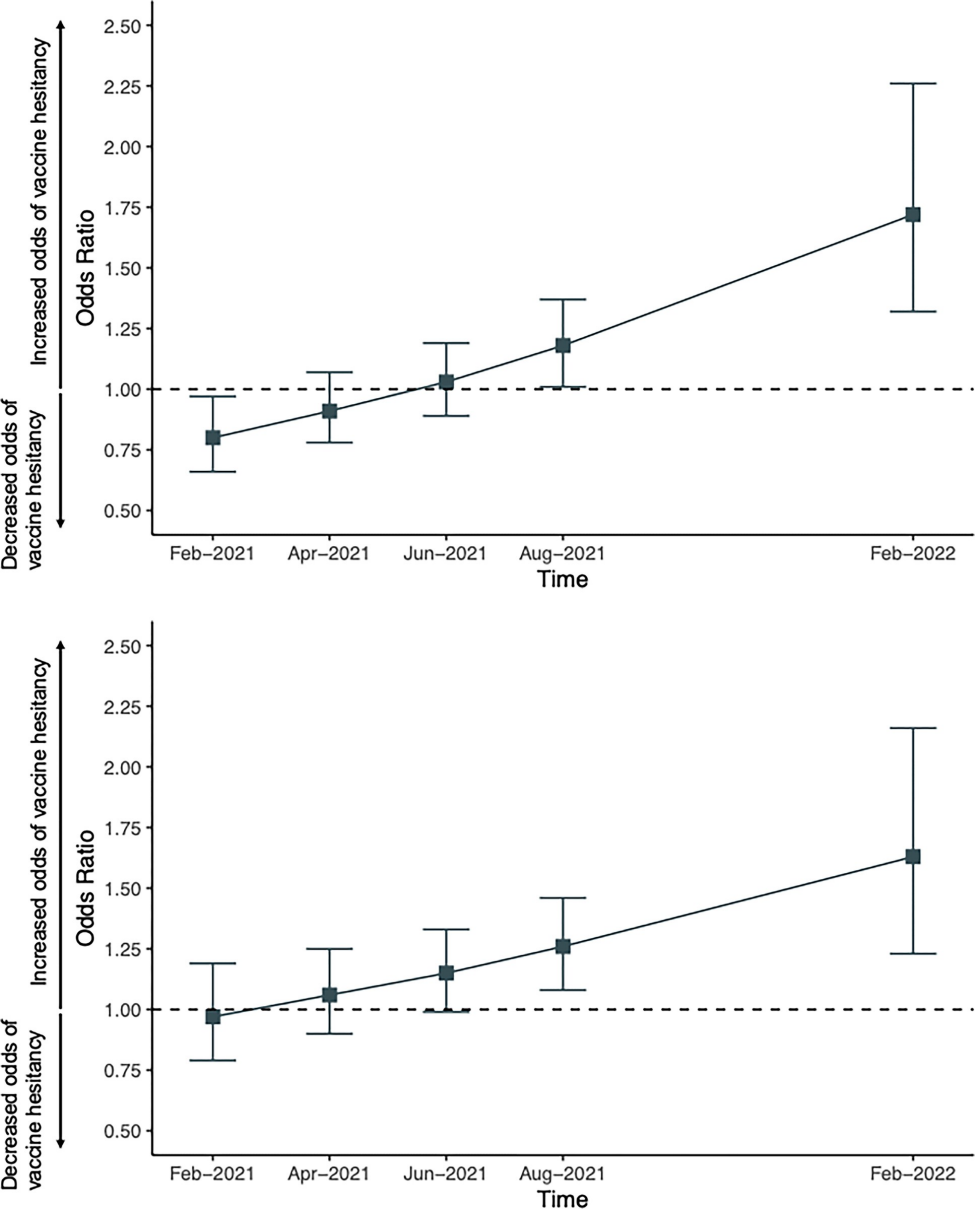
a: Mental health challenges odds ratios over time based on multivariable generalized estimating equation model. b: Substance use frequency odds ratios over time based on multivariable generalized estimating equation model.

In the present analysis, vaccine hesitancy was examined based on a convergent mixed-methods design [38]. Participants provided both qualitative and quantitative data with respect to vaccine intentions/ history (two forced-choice items), and vaccine attitudes (two open-ended questions), which were reviewed and coded by an analyst (LE). Specifically, participants were asked if they planned to receive a vaccine as soon as possible, if they had received a vaccine, their general thoughts/feelings towards the vaccine, and if their thoughts/feelings had changed since the last survey. Further, vaccine hesitancy status was identified primarily based on quantitative responses, and supplemented by qualitative responses. Based on the examined measures of vaccine hesitancy, participants were categorized into vaccine hesitant or not vaccine hesitant groups. The algorithm for determining vaccine hesitancy is presented in detail in Fig 2 of the S1 Appendix. The definition of vaccine hesitancy was designed to maximize specificity, in order to conservatively estimate vaccine hesitancy. Further, the definition was based on the merge framework described by Fetters et al. [39]. Importantly, when both qualitative and quantitative response data was available, we observed very high concordance (96%). Discordant pairs are described in the qualitative results section and could be attributed to changes in vaccine attitudes (e.g., a person who had previously received the vaccine no longer believes they are effective and regrets becoming vaccinated).

Demographic characteristics were examined across vaccine hesitancy status based on chi-squared tests. Demographic characteristics examined were gender, age, ethnic origin/background, highest level of education, born in Canada, English as a first language, urban living status, and employment/education/training status respectively. Demographic data was extracted at the timepoint when participant data was first available in all included participants.

Descriptive means (and associated 95% confidence intervals (CIs)) of mental health and substance use frequency were examined based on vaccine hesitancy status over time. Importantly, descriptive means were calculated for illustrative purposes only, and therefore statistical tests for changes in mental health or substance use scores over time or differences between groups were not examined.

The present analysis examined the relationship of mental health as well as substance use on vaccine hesitancy using generalized estimating equations (GEE) [40, 41]. GEE models were selected because we examined repeated measure longitudinal data, GEE models may account for differences in participant dropout, and GEE models can robustly estimate the impact of time-dependent covariates [40, 41]. Magnitude of effect was calculated based on odds ratios (and associated 95% CIs). Additionally, the regression model exchangeable correlation structure was determined based on an Alkaike Information Criterion best-fit analysis [40]. Further, the regression models utilized a complete case analysis methodology. In the primary regression models, domain scores (mental health challenges or substance use frequency) were examined as interaction terms with time (modelled as a continuous variable), in addition to potential confounding variables.

Potential confounding variables on vaccine hesitancy were assessed based on a bivariate GEE analysis. Specifically, each bivariate model included time (modelled as a continuous variable), the variable of interest, and their interaction. Confounding variables, modelled as an interaction term with time, were included in the primary regression model if the main effects term p-value or the interaction term p-value was less than 0.10 (an a priori threshold) [40]. The eight variables examined were: gender, age, ethnic origin/background, highest level of education, born in Canada, English as a first language, urban living status, employment/education/training status, and ever infected with COVID-19 status respectively [16, 19].

All statistical analyses were performed using SPSS version V25. Two-sided p-values < 0.05 were considered statistically significant. No adjustment for multiple hypothesis testing was performed due to the exploratory nature of the study.

Qualitative data was analyzed based on the Krippendorff methodology of content analysis [42]. An analyst (LE) reviewed all participant responses, and generated initial codes based on a theory-driven conceptualization of content areas. Main content areas were then identified after thorough discussion between the analyst and a research lead (LE and LDH), based on trends identified during the initial coding stage. Representative quotes were identified for each theme; where participant numbers are sequential and not linked to study records.

Further, we used Brunner’s pragmatic-type plot-line narrative inquiry, in order to compare trends longitudinally and cross-sectionally within and between participants [43–45]. Specifically, survey responses were organized per-person, with February 2021, April 2021, June 2021, August 2021, and February 2022 questionaries responses presented concurrently. Codes were also generated based on changes in individual vaccine attitudes over the study observation period.

Based on the content areas identified, we contextualized our results based on the vaccine hesitancy decision making matrix methodology presented by Peretti-Watel et al. [44]. Specifically, the Peretti-Watel et al. matrix analysis examines vaccine attitudes based on degree of trust in health institutions and degree of individual involvement in health decision making, corresponding to four respective vaccine attitude states: enlightened conformism, passive conformism, passive hesitancy, and enlightened hesitancy [44]. Importantly, tentative codes and content areas were co-interpreted with two youth co-researchers (JR, MP), in order to improve the trustworthiness and credibility of the qualitative analysis, as based on the Strategy for Patient-Oriented Research [46].

All participants provided electronic informed consent and Research Ethics Board approval was obtained from CAMH. As per our Research Ethics Board approval, consent was not sought from parents or guardians of participants. The present study is a youth-engaged project and was designed based on the Strategy for Patient-Oriented Research [46]. Specifically, the research question, and qualitative questions were co-developed and co-interpreted with youth co-researchers (JR, MP). Further, reflexivity was examined throughout the present study, based on discussion regarding assumptions held between analysts and the research lead, as well as discussion with youth co-researchers [46].

## Results

The study cohort included 618 participants; vaccine hesitancy data for the current study was available for 509 total participants (Fig 1 of the S1 Appendix). Specifically, the present study examined 380 participants (75%) at February 21, 434 (85%) at April 2021, 412 (81%) at June 2021, 362 (71%) at August 2021 and 448 (88%) at February 2022. Participant demographics are presented in Table 1. Over half of the participants were between the ages of 18 to 22 inclusive (60.1%). Participants identified as woman/girl (63.1%), man/boy (30.6%), or transgender or non-binary (6.3%). Additionally, over half of participant identified as Caucasian (59.2%). Vaccine hesitancy was identified in 36.0% of all participants. There were no statistically significant differences in the vaccine hesitant and non-hesitant groups based on sociodemographic characteristics.

**Table 1:** Demographic and clinical characteristics of participants.

Characteristic		Total	Vaccine Hesitant	Non- Vaccine Hesitant	Chi-squared Test p-value
		(n (%))	(n (%))	(n (%))	
Total		509 (100.0)	183 (36.0)	326 (64.0)	
Age	< 18	24 (4.7)	8 (4.4)	16 (4.9)	0.962
	18–22	304 (60.1)	110 (60.4)	194 (59.9)	
	23–28	178 (35.2)	64 (35.2)	114 (35.2)	
Gender	Man/boy	156 (30.6)	56 (30.6)	100 (30.7)	0.766
	Woman/girl	321 (63.1)	125 (68.3)	206 (63.2)	
	Transgender/non-binary*	22 (6.3)	2 (1.1)	20 (6.1)	
Ethnic origin	Caucasian	300 (59.2)	106 (58.2)	194 (59.7)	0.855
	Asian (East, South, and Southeast)	100 (19.7)	36 (19.8)	64 (19.7)	
	Multiple Ethnicities	40 (7.9)	12 (6.6)	28 (8.6)	
	Another Background**	67 (13.2)	28 (15.4)	39 (12.0)	
Highest level of education	High school diploma or less	194 (38.3)	71 (38.8)	123 (38.0)	0.928
	Greater than high school	313 (61.7)	112 (61.2)	201 (62.0)	
Not in employment, education, or training	Yes	75 (14.9)	31 (16.9)	44 (13.7)	0.387
	No	430 (85.1)	152 (83.1)	278 (86.3)	
Born in Canada	Yes	443 (87.2)	160 (87.4)	283 (87.1)	0.999
	No	65 (12.8)	23 (12.6)	42 (12.9)	
English as a First Language	Yes	460 (90.6)	161 (88.0)	299 (92.0)	0.184
	No	48 (9.4)	22 (12.0)	26 (8.0)	
Study Cohort	Clinical	215 (42.2)	78 (42.6)	86 (42.0)	0.328
	Non-Clinical	294 (57.8)	105 (57.4)	144 (58.0)	
Living Status	Urban	206 (41.0)	79 (43.6)	127 (39.6)	0.425
	Non-Urban	296 (59.0)	102 (56.4)	194 (60.4)	

Demographic data taken at the time point when participant data was first available in all included participants. Transgender/non-binary

* participants were excluded from the comparison by vaccine hesitancy status based on low observed cell sizes. Values in subgroup columns may not sum to total number of participants because of missing data. Another Background

** included the backgrounds: Black (African and Caribbean, Latin American, and Indigenous (Indigenous, First Nations, Metis) among others.

Descriptive means of mental health and substance use scores are presented in Fig 1A and 1B, respectively. In the present analysis, higher scores represented a greater perceived burden of mental health or substance use frequency Numerically, the mean mental health challenges score increased in vaccine hesitant populations and decreased in non-vaccine hesitant populations from the first time point examined (Feb-21) compared to the last time point examined (Feb-22). Additionally, numerically the mean substance use frequency score increased in vaccine hesitant populations and stayed the same in non-vaccine hesitant populations from the first time point examined (Feb-21) compared to the last time point examined (Feb-22).

Mental health challenges were observed to be protective for odds of vaccine hesitancy when COVID-19 vaccines first became available (Fig 2A and 2B). However, longitudinally, mental health challenges were observed to be associated with an increase in odds of vaccine hesitancy over time (p <0.001). Additionally, substance use was not associated with vaccine hesitancy when the COVID-19 vaccine first become available. However, over time, substance use was also observed to be associated with an increase in odds of vaccine hesitancy (p-value: 0.008).

Specifically, the odds of vaccine hesitancy decreased by 20% per 1-unit increase in mental health score (OR: 0.80 (95% CI 0.66, 0.97)) at the start of the study (Feb-21) and increased by 72% per 1-unit increase in mental health score (OR: 1.72 (95% CI 1.32, 2.26)) one year later (Feb-22) (Table 2). Further, the odds of vaccine hesitancy decreased by 3% per 1-unit increase in substance use score (OR: 0.97 (95% CI 0.79, 1.19)) at the start of the study (Feb-21) and increased by 63% per 1-unit increase in substance use score (OR: 1.63 (95% CI 1.23, 2.16)) one year later (Feb-22) (Table 3).

**Table 2:** Mental health challenges multivariable generalized estimating equation logistic regression model.

Coefficient	21-Feb	21-Apr	21-Jun	21-Aug	22-Feb	Main Effects p-value^(^^a^^)^	Interaction p-value^(^^b^^)^
Mental Health Score	0.80 (0.66, 0.97)*	0.91 (0.78, 1.07)	1.03 (0.89, 1.19)	1.18 (1.01, 1.37)*	1.72 (1.32, 2.26)*	0.003	<0.001
Age	0.95 (0.88, 1.03)	0.97 (0.91, 1.04)	0.99 (0.93, 1.06)	1.02 (0.95, 1.09)	1.08 (0.98, 1.20)	0.119	0.034
Ethnicity: Caucasian	Reference						
Ethnicity: Another Background^(^^c^^)^	1.09 (0.76, 1.59)	1.04 (0.74, 1.45)	0.99 (0.71, 1.36)	0.93 (0.67, 1.31)	0.80 (0.49, 1.29)	0.506	0.252

Odds ratios less than 1 indicate lower odds of vaccine hesitancy, odds ratio of greater than 1 higher odds of vaccine hesitancy.

Confidence intervals and p-values were calculated based on alpha: 0.05.

* Indicates a significant odds ratio.

(a) Main Effects p-value: Testing if the overall relationship between the coefficient of interest and vaccine hesitancy is significant.

(b) Interaction p-value: Testing if the relationship between the coefficient of interest and vaccine hesitancy changes over time (based on likelihood ratio tests).

(c) Pooled ethnic backgrounds included were: Asian (East, South, and Southeast), Multiple Ethnicities, Black (African and Caribbean, Latin American, and Indigenous (Indigenous, First Nations, Metis), and another background.

**Table 3:** Substance use multivariable generalized estimating equation logistic regression model.

Coefficient	21-Feb	21-Apr	21-Jun	21-Aug	22-Feb	Main Effects p-value^(^^a^^)^	Interaction p-value^(^^b^^)^
Substance Use Score	0.97 (0.79, 1.19)	1.06 (0.90, 1.25)	1.15 (0.99, 1.33)	1.26 (1.08, 1.46)*	1.63 (1.23, 2.16)*	0.290	0.008
Age	0.97 (0.90, 1.05)	0.99 (0.92, 1.05)	1.00 (0.93, 1.06)	1.01 (0.94, 1.08)	1.05 (0.94, 1.16)	0.410	0.250
Ethnicity: Caucasian	Reference						
Ethnicity: Another Background^(^^c^^)^	1.06 (0.71, 1.57)	1.01 (0.70, 1.45)	0.96 (0.68, 1.37)	0.92 (0.64, 1.32)	0.80 (0.48, 1.33)	0.404	0.299

Odds ratios less than 1 indicate lower odds of vaccine hesitancy, odds ratio of greater than 1 higher odds of vaccine hesitancy.

Confidence intervals and p-values were calculated based on alpha: 0.05.

* Indicates a significant odds ratio.

(a) Main Effects p-value: Testing if the overall relationship between the coefficient of interest and vaccine hesitancy is significant.

(b) Interaction p-value: Testing if the relationship between the coefficient of interest and vaccine hesitancy significantly changes over time.

(c) Pooled ethnic backgrounds included were: Asian (East, South, and Southeast), Multiple Ethnicities, Black (African and Caribbean, Latin American, and Indigenous (Indigenous, First Nations, Metis), and another background.

Confounding variables included in the primary regression models were age and ethnicity. The results of the bivariate analysis are presented in Table 1 of the S1 Appendix.

Content areas identified included vaccine safety perceptions, COVID-19 risk perceptions, personal experience, personal autonomy, parental/partner beliefs, social norms, and accessibility. Additionally, some participants discussed trends regarding the impacting of parental and intimate partner beliefs, trust of institutions, and trypanophobia with respect to vaccine hesitant beliefs. Importantly, we identified these content areas may be associated with positive, uncertain, and negative vaccine attitudes. The heterogeneity of the identified content areas with respect to vaccine hesitancy, both within the population as well as within individual participants:

"Scary, weird, depressing, good, odd, sad, stupid, smart, all of that. I don’t know, I just want it to end." (1)

Participants identified concerns with respect to vaccine safety including infertility, modification to DNA, myocarditis, and filler chemicals in the vaccine.

"[The vaccine is] synthetic and I’m afraid it [will] distort my DNA. I don’t want my kids to have any mental/physical problems right from birth." (2)

Additionally, participants were concerned with potential long-term side-effects. Some participants cited short clinical trials and accelerated approval as potential evidence for vaccine safety uncertainty.

"Very short clinical trial, long term effects are not yet know. First mRNA vaccine to hit the market, so this is another unknown. Animal trials skipped, not tested on children. Overall, does not sound like something I trust." (3)

In contrast, many youth were initially concerned regarding vaccine safety at the start of the study (Feb-21) and adopted a ‘wait and see’ approach. Importantly, many participants of them described believing the vaccine was safe later in the study based on the large number of people who received it without side-effects.

"I initially didn’t want to get it because I heard negative things about it (such as it causes infertility), but then I changed my mind because I want the pandemic to end." (4)

Many participants reported feeling they were at a lower risk of COVID-19 infection or complications from COVID-19 infections based on their young age, absence of co-morbidities, and occupation.

"Was less eager to get it before, for no real reason in particular, just general hesitancy and the feeling I didn’t need it due to being young and relatively healthy." (5)

A small number of participants stated they believed COVID-19 was a hoax and posed no risk: "I think it’s all fake [to be honest]" (208). These beliefs were often also discussed in combination with concerns regarding the perceived low vaccine effectiveness.

Conversely, concerns regarding high COVID-19 risk were often discussed as motivating factors to receive vaccines as soon as they become available.

"Can I get it right now? I believe that it has gone through sufficient testing. I believe that a vaccine is the only possible way of out this nightmare." (6)

Many participants commented on how personal experience with COVID-19 increased vaccine hesitancy. For example, one participant remarked that their personal experience with COVID-19 after getting vaccinated reduced their trust in the vaccine efficacy:

"I don’t believe it works anymore. I am double vaxed and still got covid. My boyfriend is unvaxed and never got it from me, despite being around me while I was infected. His entire family is all double or triple vaxed and every one of them also got covid." (7)

In comparison, some youth expressed that personal experience with COVID-19 reinforced their beliefs that vaccines were effective and necessary.

"A little concerned because my dad’s girlfriend went to the hospital with heart issues potentially due to the vaccine. But I’m convinced that I still need boosters to be safe and participate in society." (8)

In general, based on the narrative plot-line identification analysis, we identified that discussion with respect to COVID-19 personal experience was relatively uncommon at the start of the study and increased over the pandemic.

Many participants with vaccine hesitant beliefs also reported concerns regarding personal autonomy based on vaccine mandates.

"Still think it violates the principle of bodily autonomy since we cannot have informed consent fully until there is long term outcome data available." (9)

Additionally, some youth articulated they only received a vaccine because of vaccine mandate policies, and they therefore regret getting vaccinated.

"Never wanted to get vaccinated and yet I did to attend class. Now the vaccination passport is ending and I regret getting vaccinated at all." (10)

In comparison, many participants reported feelings of social responsibility outweighing personal autonomy.

"It’s the responsibility of everyone to get vaccinated for herd immunity." (11)

Some participants discussed conflicts regarding personal and parental/ intimate partner vaccine attitudes. Specifically, youth commented on how even when they wanted to receive vaccines, parental vaccine hesitant beliefs were a barrier or source of uncertainty:

"I’d get [the vaccine]. Not sure if I’d be allowed to though [because] of my parent’s concerns on the vaccine’s side effects." (12)

In comparison, youth with vaccine hesitant beliefs also reported feeling coerced into receiving the vaccine by non-vaccine hesitant parents.

"I didn’t want to get [the vaccine], was forced to so I could live with my parents after [a] break up. I still think it’s a violation of my right to bodily autonomy." (9)

Some youth reported distrust of governmental and pharmaceutical institutions throughout the study. Specifically, some participants were concerned that the government was not providing accurate health information. For example, one participant stated:

"Don’t really trust the government, believe my immune system knows how to heal itself. […] Makes me not want to get it, makes me worried about my family members who got it." (14)

Some youth also discussed concerns relating to governmental policies creating economic and racial barriers to vaccine accessibility: "It’s going to be hard to get for poor people; the government prioritizes the wealthy" (15). Some youth were concerned that booster vaccinations were a method for pharmaceutical corporations to profit from the pandemic:

"I don’t know if the boosters beyond the 3rd dose are legit or just a way for big pharma to cash in." (16)

In comparison, many participants commented they felt increased trust in health institutions as more vaccines became available and perceptions of vaccine safety increased.

"I’ve gradually become less concerned with our government bungling their application of it as supply has ramped up, and less concerned with potential unknown long-term side effects now that people have had it for around a year.” (17)

Additionally, some participants reported high trust in health institutions throughout the study.

"I have always believed in vaccines and they are safe and necessary to protect our population.” (18)

Fear of needles was reported in a small subset of youth in the study. Some participants with vaccine hesitant beliefs discussed fear of needles as preventing them from receiving the vaccine.

"I understand why some people want to get it, but I don’t. I don’t take any medications of any kind and I can’t stand needles." (19)

Additionally, participants also reported the potential need for additional booster vaccines was extremely stressful based on their fear of needles:

"Kinda disappointed that I may have to get a third vaccination. I am very, very scared of needles so it is extremely stressful for me." (21)

**Fig 3:**
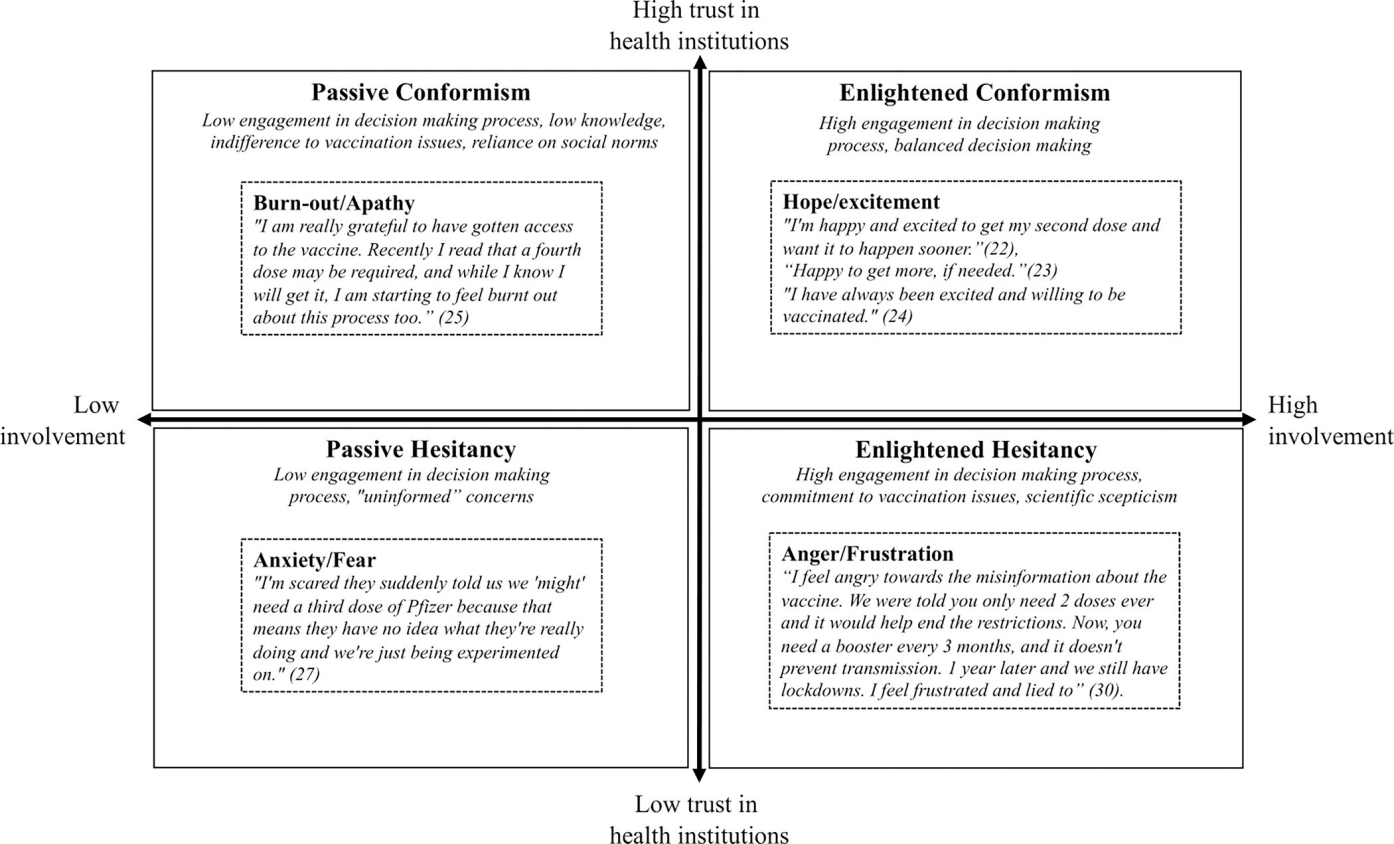
Illustration of Peretti-Watel et al. vaccine hesitancy decision making matrix analysis. Dashed boxes represent mental health states that may have changed vaccine attitudes during the pandemic. The quadrant of the dashed box illustrates the vaccine attitude held at the end of the study (Feb-22), that may be partially attributable to the specified mental health state. High and low involvement reflects individual involvement in health decision making.

In the vaccine decision making matrix analysis, we identified that mental health states were often discussed by participants as reasons for changes in vaccine hesitancy status. Specifically, we identified frequent pathways related to mental health and vaccine attitudes included hopefulness/excitement, burn-out/apathy, anxiety/fear and anger/frustration. Additionally, we identified changes in vaccine hesitancy status were frequently discussed with respect to booster vaccinations attitudes. Importantly, the identified mental health related pathways are not necessarily mutually exclusive. The results of the vaccine decision making matrix analysis are visually summarized in Fig 3.

In the present analysis, feelings of hopefulness and excitement were identified as being associated with changes from passive and enlightened hesitancy to enlightened conformism. Specifically, some youth who reported being concerned about potential side effects on the Feb-21 survey, reported excitement regarding vaccines and optimism on the state of the pandemic in Jun-21 and beyond. For example, one youth stated:

"I’m happy and excited to get my second dose and want it to happen sooner.” (22)

Additionally, participants who reported being hopeful or excited also changed vaccine attitudes less frequently, compared to those who reported feeling distraught or hopeless. For example, some participants who reported hopefulness on their Feb-21 survey, continued to report hopefulness one year later:

“I am very excited about it and want it as soon as possible.” (23; Feb-21), “I have always been excited and willing to be vaccinated.” (23; Feb-22)

Burn-out was identified as being associated with changes from enlightened conformism to passive conformism. Specifically, some youth who were initially excited to receive the vaccine as soon as possible, stated that the potential need for additional booster vaccinations felt exhausting a year later.

"I am really grateful to have gotten access to the vaccine. Recently I read that a fourth dose may be required, and while I know I will get it, I am starting to feel burnt out about this process too.” (25)

Additionally, some youth reported feeling apathetic and that they no longer cared about vaccinations a year after vaccines became available, changing from passive conformism to passive hesitancy:

“I don’t know that I really care about it anymore. […] I only really got [vaccinated] because it was mandated.” (26)

Furthermore, mental health-related challenges including anxiety/fear were associated with changes from passive conformism to passive hesitancy. Some youth reported anxiety with respect to booster vaccinations lowered their trust in health institutions.

"I’m scared they suddenly told us we ’might’ need a third dose of Pfizer because that means they have no idea what they’re really doing, and we’re just being experimented on.” (27)

Additionally, some youth who initially reported they were excited to receive the vaccine reported fear relating to side-effects and vaccine efficacy in follow-up surveys. Illustrating a potential change from active conformism to passive conformism, one participant stated:

“[I’m] scared about the potential of needing booster shots because I experienced very strong and debilitating aftereffects of the shot. So, while I feel getting the second shot was the right choice, it was also a traumatic event because I was effectively bedridden for a day extremely unsure of when the symptoms would stop.” (28)

Some youth reported feelings of anger/frustration associated with changes from passive conformism to enlightened hesitancy. Specifically, some participants who reported only receiving the vaccine based on government mandates reported decreased trust in health institutions over time, potentially increasing the propensity for passive and active hesitancy.

"It’s bullshit. 3 doses and people are still getting the virus. Something tells me it is all a lie." (29)

In comparison, some youth who initially reported they wanted to receive the vaccine as soon as possible later reported they felt frustrated and lied to by the government based on the need for booster vaccinations.

“I feel angry towards the misinformation about the vaccine. We were told you only need 2 doses ever and it would help end the restrictions. Now, you need a booster every 3 months, and it doesn’t prevent transmission. 1 year later and we still have lockdowns. I feel frustrated and lied to.” (30)

## Discussion

The present study examined the relationship between mental health and COVID-19 vaccine hesitancy in youth during the initial year of vaccine availability of the COVID-19 pandemic. Increases in mental health challenges and substance use frequency were associated with increases in odds of vaccine hesitancy over the pandemic. Importantly, mental health challenges were associated with lower vaccine hesitancy when vaccines first become available. However, one year after vaccines first became available this relationship changed direction and mental health challenges were associated with higher vaccine hesitancy. Additionally, the results of the Peretti-Watel et al. (2015) matrix analysis identified that mental health states may represent potential pathways with respect to changes in vaccine attitudes over the pandemic. Further, changes in vaccine attitudes in many participants were discussed with respect to the potential need for additional booster vaccinations to preserve COVID-19 immunity.

Importantly, the observed change in direction of the impact of mental health challenges with respect to vaccine hesitancy may be interpreted based on time perspective and mental health challenges [47]. Specifically, time perspective research suggests that persons with anxiety symptoms may be more prone to look at their future with worry and negative anticipation, and persons with depressive symptoms may be more prone to look at their past with hopelessness and negative rumination [47–49]. Therefore, anxiety symptoms may have driven increases in vaccine acceptance early in the pandemic based on fears of COVID-19 infection, as youth anxiety may have led to positive anticipation of the vaccine as a solution. However, later in the pandemic, depressive symptoms may have driven decreases in vaccine acceptance, as pandemic burnout and hopelessness may have been associated with rumination about negative aspects of the pandemic and COVID-19 vaccines, such as concerns regarding perceived limitations of vaccine efficacy. These potential pathways may be supported by prior literature suggesting that COVID-19 related anxiety symptoms were associated positively with vaccine acceptance when vaccines first became available, and depressive symptoms were associated with vaccine hesitancy, after vaccines had been available for 6-months [50, 51]. Future research may consider examining the long-term impact of anxiety and depressive symptoms with respect to COVID-19 vaccine hesitancy. The results of the present study may be further contextualized based on the health belief model [52]. Specifically, concepts identified in the present review based on a health belief model framework included perceived susceptibility, perceived risks, perceived risks, and perceived barriers [52]. Therefore, the results of the present study suggest health belief model-based interventions may be applicable to addressing COVID-19 vaccination hesitancy in youth populations.

Prior studies in general populations have suggested modestly higher vaccine hesitant attitudes in women compared to men, which attenuated over the pandemic. In contrast, prior studies in youth populations did not identify significant differences in vaccine attitudes by gender [53]. In the present study, gender was not significantly associated with vaccine attitudes in the examined regression models. However, in the qualitative analysis, some women discussed concerns with respect to the vaccine putatively causing infertility as well as mental or physical disabilities in their future children. Importantly, prior literature has suggested these attitudes may be attributed to the male bias in vaccine development and public health campaigns [53]. Specifically, the omission of menstrual and reproductive health outcomes in early COVID-19 vaccine trials may have reduced trust in the healthcare system among women [53, 54]. The results of the present study may suggest that vaccine hesitancy among women, based on reproductive health concerns may exist in some youth. Further, the unique experiences and mental health challenges of transgender/non-binary youth within this cohort have been previously well described in a qualitative longitudinal study [55]. Specifically, we identified losses with gender-diverse communities during COVID-19 may further isolate queer people from access to healthcare and therefore access to COVID-19 vaccines [55].

In comparison to prior COVID-19 vaccine hesitancy literature, we observed demographic characteristics including ethnicity, and education were not significantly associated with vaccine hesitancy [28]. Additionally, prior COVID-19 infection was also observed to not be significantly associated with vaccine hesitant attitudes or changes in vaccine attitude, as we expected. Importantly, the majority of literature examining variables related to COVID-19 vaccine hesitancy is based on adult populations [2, 3, 7–10, 56]. Therefore, the results of the present analysis may potentially support the observations of Willis et al., that conventional sociodemographic characteristics associated with vaccine hesitancy in adults (e.g., race/ethnicity, and education) may not be associated with vaccine hesitancy in youth populations [57]. Further, in contrast to the heterogeneous results examined in prior literature, we observed the direction of association between mental health challenges and vaccine hesitancy changed over the pandemic. Therefore, the prior cross-sectional studies may have identified heterogeneous magnitude and directions of association potentially because they examined discrete time-points over the duration of the pandemic [8, 17, 28].

In general, the present study identified moderately similar rates of COVID-19 vaccine hesitancy compared to those reported in prior literature, where COVID-19 vaccine hesitancy has been estimated to be approximately 58% in youth populations in the USA and 30% in the general population in the UK [9, 57]. Importantly, discussion in literature exists with respect to the large differences in vaccine hesitancy rates observed even in similar demographic populations across studies [9]. Specifically, because prior studies have utilized different methods of defining vaccine hesitancy, concerns have been discussed regarding potential uncertainty in estimating the true underlying rates of vaccine hesitancy [2, 3, 9, 14, 15, 27, 58]. For example, in the present analysis we identified some disparities between participant vaccine attitudes and reported vaccine behaviours based on the mixed-methodology analysis. Specifically, some participants reported receiving two-doses of the vaccine, while still maintaining strong vaccine hesitant attitudes and regret for receiving the vaccine. Therefore, future research may consider examining the impact of the definition of vaccine hesitancy and differences between vaccine attitudes and behaviours as a potential source of uncertainty when estimating COVID-19 vaccine hesitancy.

A prior study of US youth identified parental and peer attitudes were strongly associated with youth vaccine acceptance [59]. The present study also identified parental, intimate partner, and cultural beliefs impacted youth vaccine attitudes. Specifically, some youth reported they wanted to receive the vaccine, but were concerned how their parent or partner may react. Further, because many young people moved back to their parental home due to lockdowns and public health measures, the impact of parental vaccine attitudes may have increased during the pandemic. Therefore, the results of the present study support the conclusion that family-based interventions have been suggested as an effective method of increasing ongoing COVID-19 booster vaccine update among youth [59, 60]. In comparison, a study of US Latinx parents and youth reported youth vaccine hesitancy was not predicted by parental vaccine hesitancy (however, parental vaccine hesitancy was predicted by youth vaccine hesitancy) [61]. Importantly, in the context of the present study, these results highlight the importance of intersectionality within public health vaccination campaigns, particularly those including racialized youth populations [61].

The present study observed a period of rapid public health policy changes including stay-at-home orders, social distancing measures, and “vaccine passport” programs [62]. Therefore, the results of the present study may have implications with respect to public health policy and practice. Specifically, the results of the qualitative analysis suggest that the “vaccine passport” program in Ontario (September 22st 2021 to March 1st 2022), may not have resulted in improved COVID-19 vaccine attitudes in youth with mental health or substance use challenges [62]. Concerns in literature have recently been presented regarding the potential long-term impact of the COVID-19 pandemic with respect to trust in health institutions and attitudes towards other vaccines [63, 64]. For example, as discussed by Gencer et al., COVID-19 vaccine hesitancy may potentially result in increased vaccine hesitant attitudes with respect to all vaccines in pregnant women [63]. Therefore, the increase in vaccine hesitant attitudes identified in the present study, may potentially result in subsequent lower vaccination rates in the children of parents who experienced the COVID-19 pandemic as youth with mental health challenges. Importantly, future research may consider examining the potential long-term impacts of COVID-19 vaccine hesitancy with respect to vaccine attitudes, in youth with mental health challenges [64].

Importantly, the lower odds of vaccine hesitancy associated with mental health challenges observed when vaccines first become available, may also potentially be based on prior positive experiences with health care systems by youth with mental health challenges. Specifically, participants with higher trust in health care systems based on personal experience may report more hopeful and positive vaccine attitudes because they may have developed relationships with their health care providers or may have access to coping strategies and support systems, among other potential contributing factors [8, 16, 65]. In comparison, people with mental health challenges and vaccine hesitant attitudes may potentially feel mental health care services may be inaccessible because they may be concerned about judgement from health care providers, among other potential concerns [4]. This potential trend may therefore result in increased mental health challenges and disparities based on vaccine attitudes in youth. Therefore, future research may also consider examining the potential impact of mental health care accessibility with respect to vaccine hesitancy in this population [66].

Overall, the results of the present study suggest that vaccine hesitancy increased over the COVID-19 pandemic in youth with mental health and substance use challenges. Additionally, we observed that vaccine hesitant attitudes in the later-stages on the COVID-19 pandemic may have been driven by concerns with respect to the efficacy and necessity of COVID-19 booster vaccines. Importantly, bidirectional influences between mental health, substance use and vaccine hesitancy may have impacted results in the present study. For example, someone concerned about the dangers of COVID-19 as well as the COVID-19 vaccination, may increase their alcohol consumption in order to reduce their anxiety. Therefore, the results of the present study may not be interpreted as a causal relationship. The results of the present study may potentially have implications with respect to public policy development. Specifically, public health agencies should be aware that the current COVID-19 vaccination programs may have coincided with increased vaccination acceptance disparities for youth with mental health challenges over the first year of vaccine availability. Further, there is discussion about whether people with mental health challenges may be considered a priority vaccination group, in order allow this population earlier access to booster vaccines [27, 32]. However, the present study identified that although participants cited concerns regarding vaccine availability when vaccine first became available, increases in vaccine hesitant attitudes was the main determinant for participants not wanting to receive booster vaccines. Therefore, priority status alone may not be sufficient to increase vaccination rates in youth with mental health challenges, and public health agencies may need to consider targeted programs for youth with mental health challenges, in order to reduce vaccination disparities [26]. However, because the present study cohort was not representative of the general Canadian youth population, it is unclear as to how these results may be interpreted with respect to racialized youth communities and transgender/non-binary youth. Therefore, in order to potentially increase the feasibility, youth-friendliness and ecological validity of programs and policy, future research and public health programs may potentially consider engaging with young people in designing and targeting vaccine promotion campaigns that take their concerns into account, and to reach youth with a diversity of concerns and experiences, based on youth engagement and Patient-Oriented Research strategies [46].

## Limitations

The present study has limitations. Surveys were conducted exclusively online, and therefore may have been more likely to reach youth with consistent internet use, therefore potentially reducing the likelihood of reaching the most vulnerable youth without internet access. Additionally, bias based on potential cohort attrition may be a limitation, although (82%) of included participants responded to 3 or more of the 5 time points. However, differential participant dropout by unmeasured confounders over time may also bias the results of the present study. Future studies may consider carefully examining the potential impact of attrition bias in longitudinal cohorts examining mental health in youth populations. Importantly, because the population examined was not representative and some diverse youth may not have been included, the results may not be generalizable to youth in Canada as a whole. Further, the primary regression analysis utilized a GEE model, and therefore the associated probabilities may only be interpreted as population averages [41]. Importantly, because the population examined was not representative and some diverse youth may not have been included, the results may not be generalizable to youth in Ontario or Canada as a whole. Therefore, future studies may consider validation the results of the present study in representative cohorts. Additionally, given the relatively small number of racialized participants enrolled in the present study, we were only able to conduct regression analyses on Caucasian and pooled another background groups. However, prior literature has suggested racial disparities exist between some communities (e.g., Black communities) with respect to COVID-19 mortality and stigma, and well as mental health challenges [1]. Additionally, the present study pooled gender status of woman/girl and transgender/non-binary in the regression analysis, based on the relatively small number of transgender/non-binary participants. Because transgender/non-binary people have been suggested to experience unique challenges during the COVID-19 pandemic [35], future studies may consider examining vaccine hesitancy in this population. Therefore, the results of the present analysis may be generalized to minority populations very cautiously [1]. Further, the present study recruited participants from existing clinical and non-clinical studies led by the Centre for Addiction and Mental Health in Ontario, Canada. Therefore, recruitment bias may impact the results, because the examined population may experience more severe mental health and substance use challenges, compared to the general population. We suggest future research may consider validating the results observed in the present study within the general population. Additionally, because the present used self-reported data, recall bias may potentially result in over- or underestimation of substance use and mental health status. Further, social desirability response bias may have resulted in youth underreporting mental health challenges or substance use. Therefore, in the present analysis, we would expect response bias to bias results towards the null (no association between mental health or substance use and vaccine hesitancy). Additionally, potentially confounding variables such as peer influence, parental attitudes and access to healthcare may impact the relationships observed. Therefore, the results of the present study should be interpreted carefully. The present study also has several strengths. Specifically, the present study prospectively and longitudinally examined vaccine hesitancy, mental health, and substance use during the first year of COVID-19 vaccine availability in Canada. Additionally, the present study implemented youth engagement strategies, and was co-developed and co-interpreted with youth co-researchers [46].

## Conclusions

The present study suggested that increases in mental health challenges and substance use were associated with increases in COVID-19 vaccine hesitancy in youth populations over the COVID-19 pandemic. Additionally, we identified that booster vaccines and negative mental health states were commonly discussed with respected to increases in vaccine-hesitant beliefs. Therefore, health agencies should be aware of the potential the impact of mental health challenges and substance use frequency on vaccine hesitancy in youth populations when designing COVID-19 vaccination programs and policy.

## Supporting information

S1 Appendix (DOCX)
