# Supplementary material for: Relationship between mental health and substance abuse on COVID-19 vaccine hesitancy in youth: A mixed methods longitudinal cohort study
Source: PLoS One. 2025 Jan 8;20(1):e0313157. doi: 10.1371/journal.pone.0313157 (PMC11709255; doi:10.1371/journal.pone.0313157)
Supplement: S1 Appendix — (DOCX) [file pone.0313157.s001.docx]

Appendix

# Relationship between mental health on COVID-19 vaccine hesitancy in youth: a mixed methods longitudinal cohort study

Figure 1: CONSORT flow diagram of included participants.

Figure 2: Mixed-methods vaccine hesitancy scoring algorithm

Table 1: Regression analyses of variables of interest and vaccine hesitancy (by time interaction)

| Model | Coefficient | 21-Feb | 21-Apr | 21-Jun | 21-Aug | 22-Feb | Main Effects p-value ^(a)^ | Interaction p-value ^(b)^ |
| --- | --- | --- | --- | --- | --- | --- | --- | --- |
| 1 | Mental Health Score | **0.826 (0.687, 0.994)*** | 0.937 (0.803, 1.093) | 1.061 (0.92, 1.224) | **1.203 (1.031, 1.403)*** | **1.75 (1.332, 2.301)*** | 0.006 | <0.0001 |
| 2 | Substance Use Score | 0.936 (0.769, 1.14) | 1.033 (0.88, 1.212) | 1.139 (0.989, 1.312) | **1.256 (1.084, 1.456)*** | **1.685 (1.287, 2.205)*** | 0.188 | 0.002 |
| 3 | Age | 0.968 (0.899, 1.042) | 0.983 (0.92, 1.051) | 0.999 (0.937, 1.066) | 1.016 (0.949, 1.087) | 1.066 (0.964, 1.178) | 0.256 | 0.091 |
|  | Gender: Man/boy | Reference |  |  |  |  |  |  |
| 4 | Gender: Pooled Woman/girl and transgender/non-binary | 0.935 (0.634, 1.379) | 0.949 (0.665, 1.356) | 0.964 (0.682, 1.362) | 0.979 (0.684, 1.4) | 1.024 (0.622, 1.688) | 0.712 | 0.740 |
|  | NEET Status: NEET | Reference |  |  |  |  |  |  |
| 5 | NEET Status: Non-NEET | 0.755 (0.527, 1.08) | **0.735 (0.544, 0.994)*** | **0.716 (0.538, 0.952)*** | **0.697 (0.509, 0.955)*** | **0.645 (0.366, 1.134)*** | 0.256 | 0.662 |
|  | Living Status: Urban | Reference |  |  |  |  |  |  |
| 6 | Living Status: Non-Urban | 0.924 (0.681, 1.255) | 0.941 (0.724, 1.222) | 0.957 (0.754, 1.215) | 0.974 (0.764, 1.242) | 1.027 (0.7, 1.506) | 0.604 | 0.676 |
|  | First Language: English | Reference |  |  |  |  |  |  |
| 7 | First Language: Non-English | 1.536 (0.858, 2.754) | 1.639 (0.973, 2.762) | **1.751 (1.075, 2.857)*** | **1.872 (1.152, 3.039)*** | **2.283 (1.196, 4.348)*** | 0.282 | 0.305 |
|  | Birthplace: Non-Canada | Reference |  |  |  |  |  |  |
| 8 | Birthplace: Canada | 1.224 (0.709, 2.111) | 1.208 (0.745, 1.957) | 1.192 (0.758, 1.875) | 1.176 (0.74, 1.87) | 1.131 (0.572, 2.235) | 0.504 | 0.849 |
|  | Education: Greater than high school | Reference |  |  |  |  |  |  |
| 9 | Education: High school diploma or less | 1.237 (0.862, 1.775) | 1.183 (0.849, 1.649) | 1.131 (0.819, 1.562) | 1.082 (0.774, 1.511) | 0.946 (0.593, 1.509) | 0.213 | 0.293 |
|  | Cohort: Clinical | Reference |  |  |  |  |  |  |
| 10 | Cohort: Non-Clinical | 0.844 (0.588, 1.211) | 0.84 (0.605, 1.168) | 0.837 (0.61, 1.148) | 0.833 (0.602, 1.152) | 0.822 (0.524, 1.289) | 0.428 | 0.915 |
|  | Ethnicity: Caucasian | Reference |  |  |  |  |  |  |
| 11 | Ethnicity: Another Background^(c)^ | 1.156 (0.801, 1.67) | 1.07 (0.767, 1.493) | 0.991 (0.72, 1.363) | 0.917 (0.66, 1.274) | 0.727 (0.456, 1.16) | 0.296 | 0.082 |
|  | Exposure: Never infected with COVID-19 | Reference |  |  |  |  |  |  |
| 12 | Exposure: Ever infected with COVID-19 | 0.948 (0.642, 1.399) | 0.897 (0.655, 1.229) | 0.849 (0.658, 1.097) | 0.804 (0.641, 1.008) | **0.682 (0.479, 0.97)*** | 0.995 | 0.274 |

Legend: Odds ratios less than 1 indicate lower odds of vaccine hesitancy, odds ratio of greater than 1 higher odds of vaccine hesitancy.

Confidence intervals and p-values were calculated based on alpha: 0.05.

* Indicates a significant odds ratio.

(a) Main Effects p-value: Testing if the overall relationship between the coefficient of interest and vaccine hesitancy is significant.

(b) Interaction p-value: Testing if the relationship between the coefficient of interest and vaccine hesitancy significantly changes over time.

(c) Pooled ethnic backgrounds included were: Asian (East, South, and Southeast), Multiple Ethnicities, Black (African and Caribbean, Latin American, and Indigenous (Indigenous, First Nations, Metis), and another background.
